# Increased rostral anterior cingulate activity following positive mental imagery training in healthy older adults

**DOI:** 10.1093/scan/nsx120

**Published:** 2017-10-23

**Authors:** Susannah E Murphy, Melissa Clare O’Donoghue, Simon E Blackwell, Anna Christina Nobre, Michael Browning, Emily A Holmes

**Affiliations:** 1 Department of Psychiatry, University of Oxford, Oxford, UK; 2 Department of Psychology, Mental Health Research and Treatment Center, Ruhr-Universität Bochum, 44787 Bochum, Germany; 3 Department of Experimental Psychology and Oxford Centre for Human Brain Activity, Wellcome Centre for Integrative Neuroimaging, University of Oxford, Oxford, UK; 4 Department of Clinical Neuroscience, Karolinska Institutet, Stockholm, Sweden

**Keywords:** positive imagery, older adults, rostral anterior cingulate cortex, cognitive training, fMRI, mental imagery, emotion

## Abstract

The ability to form positive mental images may be an important aspect of mental health and well-being. We have previously demonstrated that the vividness of positive prospective imagery is increased in healthy older adults following positive imagery cognitive training. The rostral anterior cingulate cortex (rACC) is involved in the simulation of future affective episodes. Here, we investigate the effect of positive imagery training on rACC activity during the imagination of novel, ambiguous scenarios *vs* closely matched control training. Seventy-five participants received 4 weeks of positive imagery or control training. Participants underwent a functional magnetic resonance imaging scan, during which they completed an Ambiguous Sentences Task, which required them to form mental images in response to cues describing ambiguous social events. rACC activity was positively correlated with the pleasantness ratings of images formed. Positive imagery training increased rACC and bilateral hippocampal activity compared with the control training. Here, we demonstrate that rACC activity during positive imagery can be changed by the cognitive training. This is consistent with other evidence that this training enhances the vividness of positive imagery, and suggests the training may be acting to increase the intensity and affective quality of imagery simulating the future.

## Introduction

Our ability to form positive mental images about the future may be a key aspect of mental health and well-being. The vividness of mental images about positive future events is associated with dispositional optimism in non-clinical groups (Blackwell *et al.*, 2013) and amongst adults with current major depression (Ji *et al.*, 2017). Conversely, depression and dysphoria are associated with a reduced ability to form vivid mental images of positive future events and reduced accessibility of such images (MacLeod and Byrne, 1996; Stöber, 2000; Holmes *et al.*, 2008b; Morina *et al.*, 2011; Holmes *et al.*, 2016). Anxiety is associated with increased vividness and accessibility of imagery related to negative prospective events (MacLeod and Byrne, 1996; Stöber, 2000; Morina *et al.*, 2011). Whilst many cognitive models of mood disorders have focussed on biases in information processing related to the past and present, there is a growing realisation that biases in the processing of future-oriented material may also play an important role in these disorders.

Cognitive interventions targeted at shifting biases in the processing of emotional information away from negative and towards positive information are attracting increasing focus as a potential approach to the prevention or treatment of mood disorders (Woud and Becker, 2014). One such approach is to train individuals to automatically imagine positive resolutions of ambiguous information (Holmes *et al.*, 2006, 2008c). Such positive imagery training has been shown to increase state positive affect in healthy young adult volunteers (Holmes *et al.*, 2008a, 2009, 2006; Nelis *et al.*, 2013) and dysphoric individuals (Pictet *et al.*, 2011), and in some studies decrease symptoms of depression (Lang *et al.*, 2012; Williams *et al.*, 2013; Torkan *et al.*, 2014) and potentially anhedonia (Blackwell *et al.*, 2015; Williams *et al.*, 2015) in clinically depressed patients. Within the broader area of future-oriented thinking, the mental images generated during the positive training would fall into the category of ‘episodic simulations’ (Szpunar *et al.*, 2014), as the images are of specific events. However, episodic simulations are not necessarily image-based. The instructions given to participants during the positive imagery training specifically to generate mental images of the events is motivated by research indicating that mental-imagery has a particularly strong effect on emotion (e.g. Holmes *et al.*, 2006, 2008c).

We have recently investigated the effects of such positive imagery training in healthy older adults and demonstrated that it is possible to increase the vividness of positive prospective imagery using a computer-based cognitive training approach in this population (Murphy *et al.*, 2015). Increasing the vividness of positive prospective mental imagery may have particular benefits in older adults, a group where improvements to psychological treatments are needed. There is a well-documented age-related reduction in episodic future thinking, with older adults typically producing less episodically rich imagined constructs than younger adults (Addis *et al.*, 2008; Gaesser *et al.*, 2011; Lapp and Spaniol, 2017). A long history of research has demonstrated that mentally imagining future positive events can increase motivation, effort and behaviour directed at achieving them (Cantor *et al.*, 1986; Karniol and Ross, 1996; Taylor *et al.*, 1998). Given this, and the link between optimism and a range of positive physical and mental health outcomes in older adult populations (Giltay *et al.*, 2004, 2006), positive imagery training may confer particular benefits in this population.

The ventromedial prefrontal cortex (vmPFC) and adjacent rostral anterior cingulate cortex (rACC) have been shown to play a key role in the simulation of future positive affective episodes (Sharot *et al.*, 2007; D'Argembeau *et al.*, 2008; Benoit *et al.*, 2014). For example, Sharot *et al.* (2007) reported increased activity in the rACC when participants were asked to imagine positive future events, compared with when they imagined negative future events. Similarly, activity in the vmPFC has been shown to be associated with the anticipated pleasantness of episodes imagined in response to arbitrary person/place cues (Benoit *et al.*, 2014). Interestingly, the relative level of rACC activity when imagining positive *vs* negative future scenarios has been shown to be positively associated with individual differences in self-reported trait optimism (Sharot *et al.*, 2007), suggesting that this may be a critical substrate of a bias in attention and vigilance towards positive future events and away from negative events. Consistent with this, activity in the rACC has been positively correlated with likelihood estimates for positive future events (Blair *et al.*, 2013). However, it is not clear from this current evidence the extent to which such greater or lesser engagement of the rACC during positive future imagery is a stable trait, or whether it is malleable to change.

The aim of this study was to investigate whether the increased vividness of positive mental imagery reported by older adults following positive imagery training is accompanied by altered functional brain activity in the vmPFC and rACC. These regions are known to be involved in the cognitive control of emotion, and previous studies have linked activity in these regions with an attentional positivity bias in successful ageing (Brassen *et al.*, 2011). Conversely, late life depression has been associated with rACC dysfunction (Brassen *et al.*, 2008). Therefore, any intervention that provides a means to effectively increase rACC functioning may be both a useful therapeutic target in late-life depression, and a useful approach for maintaining and promoting emotional well-being and successful ageing.

## Materials and methods

### Participants

A total of 81 older adults (aged 60–80) were recruited from the community via local media and public advertisements. Four subjects withdrew before completing the study due to bereavement, poor health and difficulty travelling to the assessment centre, and two participants did not complete the magnetic resonance imaging (MRI) scan due to claustrophobia, leaving a final sample of 75 participants. All participants were fluent in English, had normal or corrected-to-normal vision and hearing, and scored >26 on the Mini-Mental State Examination (Folstein *et al.*, 1975). Participants had no current psychiatric disorder (with the exception of two participants who met criteria for specific phobia) or neurological diagnoses and were taking no psychoactive medications. All participants provided written informed consent before taking part, and ethical approval for the study was obtained from the University of Oxford Central University Research Ethics Committee (MSD-IDREC-C1-2012-112).

### Procedure

Participants were randomly assigned to either the ‘positive imagery’ or ‘control’ training condition (see Cognitive training section). Randomisation was stratified by depression scores (Beck Depression Inventory—Second Edition; BDI-II, Beck *et al.*, 1996) to ensure that mood was balanced between training groups. On completion of the training, participants completed a battery of self-report questionnaires and cognitive assessments (reported elsewhere; Murphy *et al.*, 2015) and had an MRI scan.

#### Cognitive training

**Fig. 1.**
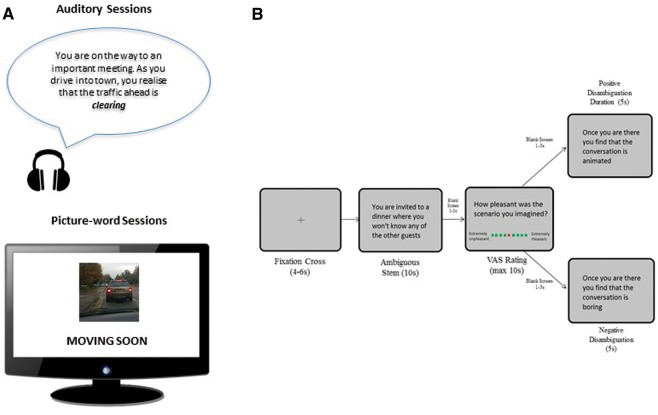
(A) Example training stimuli. Six of the training sessions were auditory (upper), in which participants listened to short descriptions of everyday situations, and six training sessions were in a picture-word format (lower), where participants were shown ambiguous photos of everyday secens paired with a word or words that resolved the scene in a positive or negative way. In the positive imagery training, all of the scenarios/scenes were resolved positively and participants were instructed to imagine themselves in the described scenario (auditory) or generate a mental image incorporating the picture and words (picture–word). In the control training, half of the scenarios/scenes resolved positively and half negatively, and participants were instructed to focus on the words and the meaning (auditory) or generate and sentence combining the picture and word (picture–word). (B) Ambiguous sentences task design. The figure illustrates two possible trials, one with a positive disambiguation, and another with a negative disambiguation relating to the ambiguous stem.

The positive imagery training was based on that previously reported (Blackwell *et al.*, 2015) and consisted of 12 sessions over a 4-week period, which encouraged the generation of positive future mental imagery. Six sessions were auditory, in which participants listened to short descriptions of everyday situations and were instructed to imagine themselves in the described scenario ‘as if actively involved and seeing it through their own eyes’. The outcome of each scenario was initially ambiguous, but all descriptions resolved positively. Six sessions were in a picture–word format, where participants were shown ambiguous photographs of everyday scenes paired with a few words that resolved the scene in a positive way. Participants were instructed to generate a mental image incorporating the picture and words. In both auditory and picture–word sessions, participants were asked to rate ‘how vividly could you imagine the described scenario?’, from 1 (not at all vivid) to 5 (extremely vivid) after each trial. Each of the 12 sessions started with reminder instructions and a practice example, followed by 8 blocks of 8 trials (64 trials per session) with self-paced breaks in between. Each session took approximately 20 min. Stimuli were not repeated; therefore, each participant was presented with 416 unique auditory stimuli and 416 unique picture–word stimuli in total across an initial practice session and 12 training sessions. Examples of auditory and picture-word training stimuli are shown in Figure 1a.

The control training differed from the positive imagery training in two ways. First, the instruction to generate active ‘imagery’ was removed and instead participants were required to process verbal aspects of the stimuli. In auditory sessions, participants were instructed to focus on the words and meanings of the descriptions, and rate ‘how difficult was it to understand the meaning of the description?’ from 1 (not at all difficult) to 5 (extremely difficult)*.* In picture–word sessions, participants were asked to generate a sentence combining the picture and words, and asked to rate ‘how difficult was it to generate a sentence?’ from 1 (not at all difficult) to 5 (extremely difficult). Second, the contingency between ambiguity and positivity was removed in the control training. To this end, half of the auditory scenarios resolved positively, whilst half had negative resolutions. Similarly, half of the pictures were paired with positive captions, whilst half had negative captions.

### MRI scan

All participants completed the MRI scan within 3 days of completing the final positive imagery/control training. During the scan, participants completed the Ambiguous Sentences Task (described below).

#### Ambiguous sentences task

Participants were shown a fixation cross for a jittered duration (4–6 s), followed by an ambiguous scenario (e.g. ‘you are invited to a dinner where you won’t know any of the other guests’), shown for 10 s. Participants were instructed to read the scenario and to generate a mental image of the scenario as if seeing it through their own eyes (field perspective), particularly focussing on how the scenario might turn out. A jittered blank screen was then displayed (1–3 s), after which participants were asked to rate how pleasant the outcome of their imagined future scenario was on a Visual Analogue Scale (VAS) from 1 (extremely unpleasant) to 9 (extremely pleasant). The VAS was presented for a maximum of 10 s, or until the participant made a button response. After a second jittered blank screen (1–3 s), participants were presented with an outcome disambiguating the initial scenario. The outcome could be either positive (e.g. ‘once you are there you find that the conversation is animated’) or negative (e.g. ‘once you are there you find that the conversation is boring’). During the 5 s presentation of the disambiguation, participants were asked to reimagine the initial scenario incorporating the new information. No behavioural response was required before the onset of the fixation cross for the next trial. The task design is shown in Figure 1b.

The task consisted of 40 novel ambiguous stems, each with a possible positive and negative disambiguation. All participants were presented with each stem once only (order randomised across subjects) and were randomly assigned 20 positive and 20 negative disambiguations. Participants were given a self-paced break every 10 trials. The stems were between 8 and 18 words long and the disambiguations were between 5 and 15 words long. The positive and negative disambiguations for each scenario were matched for length. Scenarios were presented on a dark grey background using Presentation version 16.2 software (Neurobehavioual Systems, Inc.) on a screen, which was viewed via the reflection in a mirror on the head coil by participants inside the scanner.

Before going into the scanner, participants were given task instructions, and were asked to only imagine the scenarios for as long as the words appeared on the screen*.* Participants were also given a pictorial example of the difference between imagining in field perspective (as required by this task) *vs* observer perspective. Each participant was given three practice trials to check comprehension of the instructions, with one practice trial guided by the researcher, and the remaining two completed independently.

#### Image acquisition

Participants were scanned using a 3 T Siemens TIM Trio System (Siemens, Erlangen, Germany) at the Oxford Centre for Magnetic Resonance Imaging (OCMR), using a 32-channel head coil. An EPI-BOLD contrast image with a total of 45 slices was acquired, with a voxel resolution of 3 × 3 × 3 mm^3^, repetition time = 3000 ms, echo time = 30 ms. Slice angle was set to 30° and flip angle was 87°. Cardiac and respiratory physiological data were acquired during the EPI scan, using a pulse oximeter and chest bellows, to allow for the effects of physiological noise in the functional MRI (fMRI) data to be modelled during the analysis. A fieldmap image was also acquired to correct signal distortions and compensate for signal loss in the EPI signal. Fieldmap parameters were: voxel resolution 3.5 × 3.5 × 3.5 mm^3^, repetition time = 488 ms, first echo time = 5.19 ms and second echo time = 7.65 ms. A high-resolution 3D whole-brain T1-weighted structural image was acquired for registration purposes, using an MP RAGE sequence, with voxel resolution of 1 × 1 × 1 mm^3^, repetition time = 2040 ms and echo time = 4.7 ms.

### Data analysis

Baseline demographic and mood measures were compared between groups using independent *t*-tests for continuous data and chi-square tests for categorical data. The difference in pleasantness ratings of the ambiguous stems between the two groups was analysed using an independent samples *t*-test.

Five participants (three imagery and two control training) were excluded from the imaging analysis due to cortical abnormalities observed in their T1-structrual scans, leaving a total of 70 participants (32 positive imagery training group and 38 control group).

fMRI data processing was carried out using FEAT (FMRI Expert Analysis Tool) Version 6.00, part of FSL (FMRIB Software Library, Oxford Centre for Functional Magnetic Resonance Imaging of the Brain, Oxford University, Oxford, UK, http://www.fmrib.ox.ac.uk/fsl). Pre-processing consisted of head motion correction (using MCFLIRT, Jenkinson *et al.*, 2002), brain extraction (using FSL’s Brain Extraction Tool, Smith, 2002), spatial smoothing using a Gaussian kernel of full width at half maximum 8 mm and highpass temporal filtering set at 100 s. Images were also unwarped using B-0 fieldmaps (Jenkinson, 2003, 2004). Functional data were registered to standard-space using FSL’s BBR and non-linear registration tools (Jenkinson and Smith, 2001; Jenkinson *et al.*, 2002; Andersson *et al.*, 2007a,b; Greve and Fischl, 2009;).

The demeaned self-reported VAS ratings of pleasantness were included as a regressor in the first level model for the imaging analysis, and were timelocked to the onset of the ambiguous stem. The events coded by this ‘pleasantness’ regressor had the same duration as the stem presentation (10 s). Trials where no pleasantness ratings were recorded (0.61% of trials) were excluded from the analysis. There was no significant difference between the two training conditions in the number of trials excluded on this basis (*P* > 0.1).

The 5-s time period when the disambiguation was displayed on the screen was modelled as two separate explanatory variables (EVs) ‘positive disambiguation’ and ‘negative disambiguation’.

A boxcar convolved with a *γ* haemodynamic response function and its temporal derivative was used to model the data. Physiological data were processed within FSL to produce physiological noise EVs (Brooks *et al.*, 2008), which were entered as voxel-wise confound regressors in the model. Timepoints affected by large head movement that remaining after motion correction, identified by FSL’s Motion Outliers tool, were also included in the model as confound regressors.

Group level analysis was carried out using FMRIB’s Local Analysis of Mixed Effects (Woolrich *et al.*, 2004). *Z* (Gaussianised *T*) statistic images were thresholded using clusters determined by *Z* > 2.3 and a (corrected) cluster significance threshold of *P* < 0.05 (Worsley, 2001). For the identified clusters, Montreal Neurological Institute (MNI) co-ordinates provide the location of the peak voxel within the cluster, *Z*-max is the *z* score for this voxel and the *P* value is corrected at the cluster level.

## Results

The groups were well matched in terms of age, education, gender, ethnicity, marital status, employment status and baseline mood (see Table 1). There were no significant effects of training on subjective measures of depression, anxiety or affect (see Table 2).

**Table 1.** Baseline demographic characteristics for the two training groups

	Imagery	Control
	(*n* = 32)	(*n* = 38)
Age (years)	68.1 (60–80)	66.3 (60–78)
Years of education	15.9 (5–24)	17.0 (10–31)
Gender (*n* female)	20 (62.5%)	19 (50.0%)
Ethnicity (*n* white)	31 (96.9%)	38 (100.0%)
Marital status		
Married	16 (50.0%)	24 (63.2%)
Cohabiting	1 (3.1%)	3 (7.9%)
Single	15 (46.9%)	11 (28.3%)
Employment status		
Current full time	3 (9.4%)	6 (15.8%)
Current part time	4 (12.5%)	5 (13.2%)
Retired	24 (75.0%)	26 (68.4%)
Unemployed	1 (3.1%)	1 (2.6%)

Notes: Independent sample *t*-tests conducted for age, years of education. Chi-square tests conducted for gender and ethnicity. Values in parentheses represent: ‘range’ for age and years of education, and ‘percentage’ for gender, ethnicity, marital status and employment status.

**Table 2.** The effect of training on depression, anxiety and affect

	Imagery (*n* = 32)		Control (*n* = 38)		
	Pre- training	Post- training	Pre- training	Post- training	Statistics (*P*)
BDI-II	4.8 (3.4)	3.6 (3.1)	4.8 (4.3)	3.8 (4.9)	0.72
STAI (Trait)	32.4 (8.1)	30.1 (9.1)	33.0 (8.6)	32.1 (8.9)	0.21
PANAS	(*n* = 31)		(*n* = 36)		
Positive	34.3 (6.2)	35.5 (5.2)	34.4 (6.7)	35.2 (7.2)	0.94
Negative	13.9 (3.0)	12.6 (3.8)	14.2 (4.2)	13.2 (3.8)	0.73

Abbreviations: PANAS, Positive And Negative Affect Schedule; STAI, State-Trait Anxiety Inventory. Independent sample *t*-tests conducted BDI, STAI, PANAS positive and PANAS negative. Values in parentheses represent standard deviation*.* Each variable was analysed using a repeated measures ANOVA with training group as a between subjects factor (imagery, control) and time as a within groups factor (pre-training, post-training). The *P* values displayed are for the group × time interaction. PANAS data are missing for three participants due to a technical error. N.B these data have previously been reported (within a larger sample) in Murphy *et al*. (2015).

There was no significant difference in average ‘pleasantness’ rating between training groups on the ambiguous sentence task (imagery group mean rating = 6.52; control group mean rating = 6.50), as shown by an independent sample *t*-test [*t*(68) = 0.12, *P* = 0.91].

### Pleasantness ratings of ambiguous stem

**Fig. 2.**
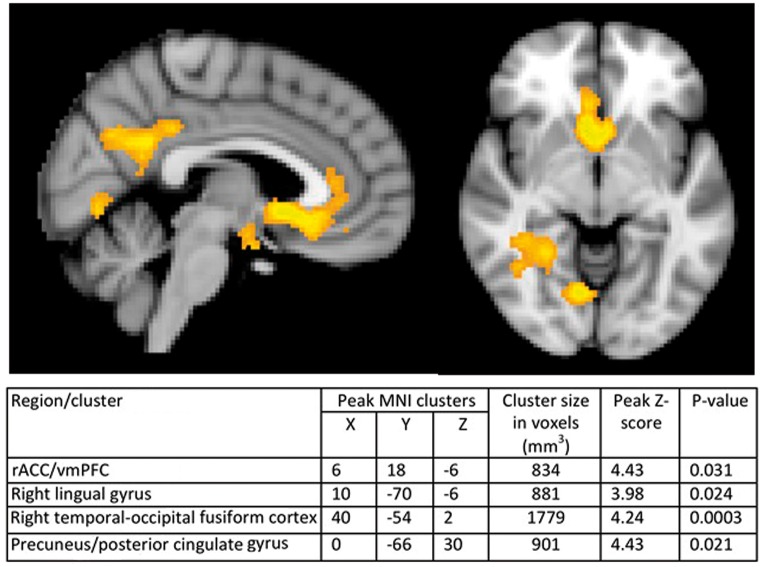
Results of whole-brain analyses showing activity significantly associated with the pleasantness regressor across all participants (cluster corrected *P* < 0.05).

**Fig. 3.**
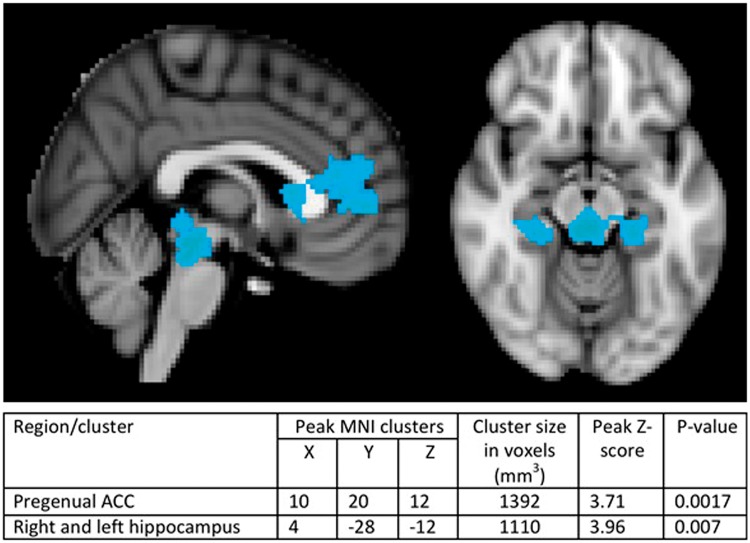
Results of whole-brain between-group analyses showing activity in the rostral anterior cingulate and the bilateral hippocampus (extending into the brainstem) was modulated by the training group.

A whole-brain analysis identified a network of regions that covaried with the pleasantness regressor (see Figure 2), with four clusters in the rostral anterior cingulate extending into the vmPFC [peak MNI coordinates: 6, 18, –6; *Z* max = 4.43; *P* = 0.031], right lingual gyrus [peak MNI coordinates: 10, –70, –6; *Z* max = 3.98; *P* = 0.024], the right temporal-occipital fusiform cortex [peak MNI coordinates: 40, –54, 2; *Z* max = 4.24; *P* = 0.0003] and the precuneus and posterior cingulate [peak MNI coordinates: 0, –66, 30; *Z* max = 4.43; *P* = 0.021]. Whole-brain between-group analysis (see Figure 3) revealed two clusters in the pregenual anterior cingulate [peak MNI coordinates: 10, 20, 12, *Z* max = 3.71, *P* = 0.0017] and the bilateral hippocampus [peak MNI coordinates: 4, –28, –12; *Z* max = 3.96; *P* = 0.007] in which there was a significant interaction between training group and the pleasantness regressor, with increased activity in the positive training group compared with the control group. Further analysis of the imagery group and control group separately demonstrated that the activity associated with increasing ratings of pleasantness in both the rostral anterior cingulate and the bilateral hippocampus was evident in the positive imagery group but not in the control group.

### Response to valence of stem disambiguation

There was increased activity in a network of areas in response to a positive disambiguation compared with a negative disambiguation, including the anterior cingulate gyrus, supramarginal gyrus, precuneus, lateral occipital cortex, hippocampus and parahippocampal gyrus. However, there was no significant effect of training group on BOLD signal in response to positive *vs* negative disambiguations. In response to negative disambiguations compared with positive disambiguations, there was increased activity in the superior frontal gyrus and the bilateral inferior frontal gyrus, but again this activity was not significantly modulated by training group.

## Discussion

The aim of the current study was to investigate the effect of positive mental imagery training on functional brain activity during the imagination of novel, ambiguous future-oriented scenarios. Activity in the rACC and vmPFC was positively correlated with the pleasantness ratings of participants’ mental images formed in response to a cue describing an ambiguous event. After 4 weeks of computer-based training, the positive imagery group showed an increase in rACC activity compared with the closely matched control training group. In addition, there was an increase in activity in the bilateral hippocampus in the positive imagery training group compared with the control training group. These effects on functional brain activity were seen in the absence of any effect of training group on the behavioural ratings of the pleasantness of the ambiguous scenarios. There was no difference between the two training groups in brain activity in response to the disambiguation of the scenarios as positive or negative.

The increased activity in the rACC and adjacent vmPFC associated with pleasantness ratings of the images formed in the current study is consistent with previous reports of a role for this region in the positive simulation of future events. Sharot *et al.* (2007) found increased activity in a similar rACC region when participants imagined positive future events compared with when they imagined negative future events. Similarly, Benoit *et al.* (2014) reported activity in the vmPFC that increased as the pleasantness of imagined future scenarios increased. Consistent with this, the present finding of increasing activity in the rACC/vmPFC as participants’ ratings of the pleasantness of the imagined scene increase adds to the growing literature suggesting that these regions play a role in coding for the value and affective quality of imagined scenarios. Such activity is consistent with this region’s broader role in the representation of the subjective value of a stimulus (Kable and Glimcher, 2007; Rushworth *et al.*, 2011; Winecoff *et al.*, 2013; Gross *et al.*, 2014). Taken with the results of the current study, there is converging evidence that activity in the rACC/vmPFC during the imagination of future events reflects the associated positive affective quality of that experience (Benoit *et al.*, 2014).

Critically, the current study demonstrates that the engagement of the rACC during positive imagery is malleable to change, and can be increased by positive imagery training. Following 4 weeks of positive imagery training, there was increased activity in the rACC associated with increasing pleasantness ratings of the ambiguous scenarios, compared with the control training group. The pleasantness regressor used in the analysis can be conceptualised as a continuous measure of value; a larger signal is seen as the distance increases between activity in response to positive image *vs* negative image. The finding that this signal is increased following positive imagery training suggests that the neural response in the rACC and hippocampus to positive compared with negative images was more distinct in those who had undergone the positive imagery training compared with the control training group. Such a finding is in line with our previous report of increased vividness of positive prospective imagery following 4 weeks of positive imagery training (Murphy *et al.*, 2015), and suggests that this training may be acting to increase the intensity and affective quality of mental imagery.

The effect of positive imagery training on rACC activity is interesting in light of the evidence to suggest that this region may not only be involved in representing the pleasantness, or subjective value, of imagery about future events, but also individuals’ bias in their expectation of such positive events occurring to themselves. Recent neuroimaging studies have suggested that such biases in the estimation of the probability of positive events occurring to oneself relative to others is related to activity in the rACC. For example, Blair *et al.* (2013) reported that activity in this region was positively correlated with participants’ likelihood estimates of positive future events occurring to themselves relative to others. This is in line with the finding that relatively increased activity in the rACC during the imagination of positive compared with negative future scenarios is positively correlated with self-reported optimism (Sharot *et al.*, 2007). The increase in activity in the rACC following positive imagery training seen in the current study is therefore consistent with the association that has been reported between the ability to generate vivid mental images of positive future events and dispositional optimism (Blackwell *et al.*, 2013; Ji *et al.*, 2017).

Interestingly, there is evidence that the rACC is involved in the positive bias, or ‘positivity effect’ that is well described in older populations, whereby there is an increased focus on positive compared with negative information (Steinman *et al.*, 2013; Reed *et al.*, 2014). For example, older adults have been shown to have specifically increased distractibility by happy faces compared with younger adults, which is associated with enhanced activation in the rACC, which in turn is associated with emotional well-being (Brassen *et al.*, 2011). Conversely late life depression has been associated with decreased recruitment of rACC during the processing of emotional stimuli compared with healthy older adults (Brassen *et al.*, 2008). In light of this, the effect of positive imagery training on rACC activity suggests that it may be a useful cognitive intervention to promote affective well-being in healthy ageing.

Positive imagery training also increased activity in the hippocampus associated with pleasantness ratings of the ambiguous scenarios. This is consistent with the idea that the positive imagery training was acting to increase the richness of representation of the positive images formed in response to the ambiguous cue. The medial temporal lobe (including the hippocampus) is thought to be critically involved in the simulation of future mental events, and a number of studies have highlighted the overlap in neural systems involved in remembering past events and imagining future experiences (Addis *et al.*, 2007; Szpunar *et al.*, 2007; Schacter *et al.*, 2012). Hippocampal damage has been shown to be associated with an impairment in imagining novel experiences (Hassabis *et al.*, 2007). Interestingly, a recent study has reported reduced engagement of medial temporal regions during the construction of future events in depressed patients (Hach *et al.*, 2014), which is consistent with the reduced episodic detail and vividness of mental imagery that is associated with this disorder (Holmes *et al.*, 2008b; King *et al.*, 2011). With the current paradigm, we do not know whether participants were imagining novel future events to resolve the ambiguous scenarios or, at least to some extent, recasting a past event. Imagined outcomes for ambiguous events in everyday will similarly likely be a combination of memories of past events and newly generated images created by drawing on memory (e.g. Addis *et al.*, 2007). However, to differentiate between these possibilities more precisely in future research other paradigms designed to increase the likelihood that novel events are simulated (e.g. Szpunar *et al.*, 2015) could be used.

Given that the positive imagery training involved repeatedly presenting ambiguous sentences followed by positive resolutions, whereas the scenarios only resolved positively on half of the control training trials in the control training (and negatively on the other half), it is perhaps surprising that there was no difference between the training conditions in the response to the later stem disambiguation. If the training had been successful in shifting participants’ expectations of positive resolutions following ambiguity, we might have expected to see a ‘surprise’ signal on trials where there was a violation of the training contingency and the stem was resolved negatively. The lack of such an effect suggests that, whilst the training was acting to increase the vividness of positive imagery, this was not positively biasing their interpretation of the ambiguous scenarios. Interestingly, this mirrors the pattern of effects that we saw at a behavioural level (Murphy *et al.*, 2015) that, whilst the positive imagery training increased the vividness of positive imagery about the future, there was no effect of this training on interpretive bias (as measured by the Scrambled Sentences Task). This is in contrast to studies in clinically depressed samples that have demonstrated an effect of the positive imagery training on measures, such as the Scrambled Sentences Task (a measure of biases in interpretation of emotionally ambiguous information, Lang *et al.*, 2012; Williams *et al.*, 2013; Torkan *et al.*, 2014; but see also Blackwell *et al.*, 2015; Williams *et al.*, 2015). It would be interesting to investigate in future studies whether there is an effect of positive imagery training on the response to the disambiguation in low mood samples, which would be consistent with these behavioural patterns.

There are a number of limitations to the current study that should be noted. First, there was no difference between the two training conditions in self-report emotional well-being or optimism (reported in Murphy *et al.*, 2015). As previously discussed, it may be that the ‘non-specific’ and social effects of taking part in the study and intervention schedule had a generalised positive effect in both conditions that outweighed any relatively more subtle effects of changes of activity in specific brain regions. Further, it may be that relatively sustained changes in rACC activity would be needed to lead to changes in relatively stable characteristics such as optimism. However, the present study provides proof-of-principle that such changes are possible via training, opening the possibility of future work to investigate potential benefits. Second, the positive training induced changes in rACC and hippocampal activity are challenging to interpret in the absence of a significant behavioural effect of training on the fMRI task. Future studies should include a behavioural measure of the vividness of the images to further clarify the relationship between the neural activity changes and the vividness of positive and negative imagery after imagery training. Finally, since only the positive imagery group practised forming mental images, it is not possible to tell whether changes observed in the current study relate to the positive nature of the images generated or repeated practice in imagery generation *per se*. However, in our previous report of the effects of this training on prospective imagery (Murphy *et al* 2015), an increase in imagery vividness in the positive imagery training group compared with the control group was only observed for positive prospective imagery, and not negative imagery, suggesting that the training did not result in generalised changes in the ability to generate imagery *per se*.

In summary, the current study demonstrated that it is possible to increase the engagement of the rACC during positive mental imagery through positive imagery cognitive training. These effects are consistent with previous reports of this training increasing the vividness of positive imagery at a behavioural level and lend weight to the idea that it is acting to increase the intensity and affective quality of imagery about the future. Taken together this supports the idea that positive imagery cognitive training may be a useful approach to examine for promoting and maintaining emotional well-being.

## Funding

This research was supported by the National Institute for Health Research (NIHR) Oxford Biomedical Research Centre based at Oxford University Hospitals Trust and University of Oxford and a Wellcome Trust Senior Investigator Award (ACN, 104571/Z/14/Z). The Wellcome Centre for Integrative Neuroimaging is supported by core funding from the Wellcome Trust (2013139/Z/16/Z). The views expressed are those of the authors and not necessarily those of the NHS, the NIHR or the Department of Health. This research was also supported by the NIHR Oxford Cognitive Health Clinical Research Facility.

*Conflict of interest*. None declared.
